# Infectious RNA: Human Immunodeficiency Virus (HIV) Biology, Therapeutic Intervention, and the Quest for a Vaccine

**DOI:** 10.3390/toxins14020138

**Published:** 2022-02-14

**Authors:** Yasemin van Heuvel, Stefanie Schatz, Jamila Franca Rosengarten, Jörn Stitz

**Affiliations:** 1 Research Group Pharmaceutical Biotechnology, Faculty of Applied Natural Sciences, TH Köln—University of Applied Sciences, Chempark Leverkusen, Kaiser-Wilhelm-Allee, 51368 Leverkusen, Germany; yasemin.van_heuvel@th-koeln.de (Y.v.H.); stefanie.schatz@th-koeln.de (S.S.); jamila_franca.rosengarten@th-koeln.de (J.F.R.); 2 Institute of Technical Chemistry, Leibniz University Hannover, Callinstraße 3-9, 30167 Hannover, Germany

**Keywords:** retroviruses, HIV, virus replication, mRNA splicing, antiretroviral therapy (ART), HIV vaccines

## Abstract

Different mechanisms mediate the toxicity of RNA. Genomic retroviral mRNA hijacks infected host cell factors to enable virus replication. The viral genomic RNA of the human immunodeficiency virus (HIV) encompasses nine genes encoding in less than 10 kb all proteins needed for replication in susceptible host cells. To do so, the genomic RNA undergoes complex alternative splicing to facilitate the synthesis of the structural, accessory, and regulatory proteins. However, HIV strongly relies on the host cell machinery recruiting cellular factors to complete its replication cycle. Antiretroviral therapy (ART) targets different steps in the cycle, preventing disease progression to the acquired immunodeficiency syndrome (AIDS). The comprehension of the host immune system interaction with the virus has fostered the development of a variety of vaccine platforms. Despite encouraging provisional results in vaccine trials, no effective vaccine has been developed, yet. However, novel promising vaccine platforms are currently under investigation.

## 1. Introduction

RNA viruses are recognized as the leading causes of human infectious diseases. Since the first discovery of infectious RNA viruses in humans in 1900, namely, the yellow fever virus (YFV) from the family *Flaviviridae*, a total of 214 human RNA viruses have been identified, to date [1,2]. Many of these viruses, such as rabies virus (RABV), poliovirus (PV), dengue virus (DENV), and measles virus (MeV), have been transmitted in humans since several hundreds of years [3,4,5,6]. In the last couple of decades, numerous human pathogenic RNA viruses have emerged by crossing the species barrier from their natural animal host to humans. These zoonotic transmissions include the Ebola virus (EBOV), Zika virus (ZIKV), severe acute respiratory syndrome coronavirus types 1 and 2 (SARS-CoV-1, SARS-CoV-2), middle east respiratory syndrome coronavirus (MERS-CoV), and of course the human immunodeficiency viruses types 1 and 2 (HIV-1, HIV-2) [7].

HIV belongs to the virus family *Retroviridae* and is grouped into the genus Lentivirus, first isolated and identified in 1983 [8,9]. The first transmission to humans most likely occurred during the past century, assumingly between 1920 and 1940. HIV originated from several zoonotic transmission events from non-human primate simian immunodeficiency viruses in Central African chimpanzees (SIVcpz; HIV-1) and West African sooty mangabey monkeys (SIVsmm; HIV-2) [10]. Since 1983, the HIV epidemic has caused an estimated 36.3 million deaths and 37.7 million people lived with the infection worldwide in 2020 [11].

The infection with HIV mostly occurs during sexual contact across mucosal surfaces. Maternal-infant exposure and shared use of needles during drug abuse can also facilitate transmission of the virus [12]. The viral tropism mainly targets T helper cells—key regulators of humoral and cellular immune responses—where most of the viral replication takes place. HIV induces the most extreme form of immune subversion caused by pathogens in humans and leads to a continuous loss of CD4^+^ T helper cells. The diminishment of the T helper cell population increasingly weakens the immune system. During the progression to the acquired immunodeficiency syndrome (AIDS), the ability to prevent infections with other pathogens collapses and causes death by opportunistic infections [10]. HIV also infects other cell types such as macrophages, dendritic cells, and resting T cell subsets. These host cells also play a pivotal role in innate and adaptive immunity. All three cell types often function as viral reservoirs harboring transcriptionally inactive proviruses. This allows HIV to establish a persisting infection and to escape from detection and eradication by immune cells and therapeutic interventions, respectively [10].

To date, antiretroviral therapy (ART) is the only available treatment of infected humans, saving several thousands of lives each year. ART relies on the combination of three or four virus replication inhibitors. However, ART does not cure from infection but limits virus replication, viral load, and thus the progression to AIDS. This transforms the formerly fatal HIV infection to a chronic disease. The required long-term treatment, however, leads to the development of multi-drug resistant viruses and is burdened with undesired adverse effects such as anorexia, nausea, vomiting, and diarrhea associated with the discontinuation of the therapy. ART is also cost-intensive. Considering increasing numbers of infected people, the costs for ART treatment will become unaffordable. Thus, a vaccine is urgently needed to fight the epidemic. However, no sufficiently potent vaccine against HIV has yet been developed [13].

This review will first focus on the genomic organization of HIV, the virion structure, and the replication cycle from virus cell entry to the egress of new infectious particles as well as the cytotoxicity of infection. An overview is provided on antiviral compounds used in ART and phase III clinical trials of vaccine candidates.

## 2. HIV-1 Structure and Replication Cycle

### 2.1. Genome and Virion Structure

**Figure 1:**
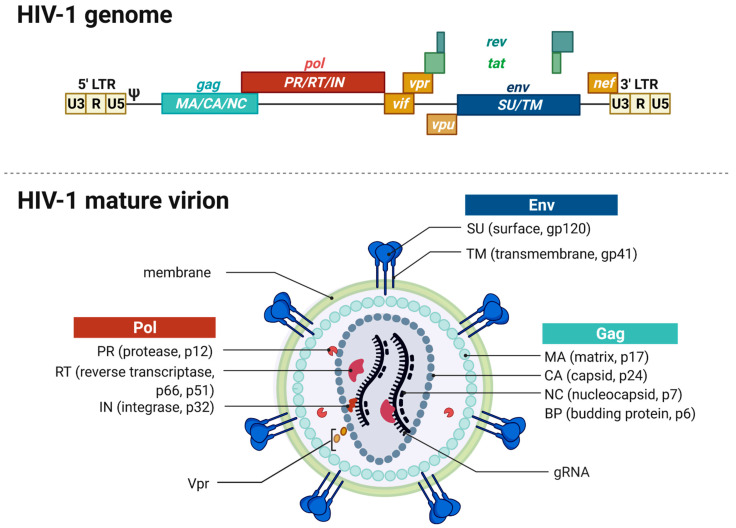
HIV-1 genome and virion structure. (**Top**) Schematic overview of the genomic organization of the HIV-1 genome encompassing the open reading frames coding for the different structural, regulatory, and accessory proteins. The dimeric, linear gRNA is ~9 kb long and flanked by the 5′- and 3′-long terminal repeats (LTRs) that contain the viral promoter and sequences required for reverse transcription, integration, and gene expression. The LTRs are distinguished into *cis*-acting regulatory elements, namely, U3, R, and U5 regions followed by the packaging signal Psi (ψ). *Gag* encodes the structural proteins matrix (MA), capsid (CA), and nucleocapsid (NC) forming the viral core. *Pol* codes for the viral enzymes protease (PR), reverse transcriptase (RT), and the integrase (IN). The *Pol* gene is followed by the two regulatory genes *rev* and *tat* and three accessory genes *vif*, *vpr*, and *vpu*. *Env* encodes the viral envelope glycoproteins—the surface unit (SU) gp120 and the transmembrane unit (TU) gp41. *Env* is followed by another accessory gene *nef*. (**Bottom**) The mature enveloped virion has a spherical shape and is enveloped by a lipid bilayer membrane derived from the host cell containing 7–35 envelope glycoproteins trimers. The inner layer of the membrane anchors the Gag-derived MA proteins and also harbors Vpr and PR. The capsid is found within the center of the virion and contains the two copies of gRNA, RT, and IN. The gRNA is stabilized by the NC proteins.

The RNA genome (gRNA) of HIV-1, with approximately 9 kb, is considerably small. However, it contains all necessary information to synthesize all 15 proteins needed for replication and assembly of new virions in the infected host cells [14,15]. The viral genome encapsulated in virions consists of a dimer of single stranded positively sensed gRNAs. The different open reading frames (ORFs) are illustrated in Figure 1, except for the ORF encoding the antisense protein, yet uncharacterized for its role in the replication cycle [14,16]. The genome encompasses nine different ORFs and some of the viral genes overlap, thus enabling the encryption of many proteins within a limited coding capacity. The genome is flanked by the long terminal repeats (LTRs). They contain the essential information—including the viral promoter—for gene expression, integration, and reverse transcription and are divided into the U3, R, and U5 elements [10]. The *cis*-acting regulatory element U3 is divided into a modulatory, an enhancer, and a basal region and contains three binding sites for splice factors as well as two binding sites for host cell transcription factors, e.g., Nuclear Factor-κB (NF-κB). The R element contains the *trans*-acting responsive region (TAR), forming a RNA stem-loop structure that plays an important role in viral replication, i.e., the activation of transcription [17,18]. The U5 element contains the polyadenylation signal (poly A) and regulatory regions for reverse transcription. The U5 element is followed by the primer binding site (PBS), the dimerization initiation signal (DIS), and the major splice-donor site (D1), all not shown in Figure 1. The packaging signal Psi (ψ) mediates the packaging of the viral gRNA [19]. The consecutive *gag* gene encodes the structural viral core proteins. The precursor protein p55-Gag is processed by the viral protease during virion maturation into the subunits matrix (MA), capsid (CA) and nucleocapsid (NC) proteins. The *pol* gene encodes the subunit viral enzymes protease (PR), reverse transcriptase (RT), and integrase (IN), also originating from a precursor protein upon viral protease-mediated cleavage. The third structural gene *env* encodes the two envelope glycoproteins gp120-SU (surface unit) and the gp41-TM (transmembrane unit). The *pol* gene is followed by the two regulatory genes *rev* and *tat* as well as four accessory genes *vif*, *vpr,* and *vpu*. Tat and Rev are indispensable for viral replication, accumulate within the host cell nucleus and bind to their cognate mRNA structures, namely, the Rev-responsive element (RRE) and TAR. Rev is an important nuclear export factor that mediates the transport of partially spliced and unspliced viral mRNAs into the cytoplasm. Tat is a strong transcriptional activator [20,21,22]. Vif, Vpr, and Vpu influence the rate of virus particle production. The accessory *nef* gene at the end of the gRNA elevates HIV infectivity and downregulates several host cell proteins including CD4 and the major histocompatibility complex I (MHC I) [23]. Moreover, Vif, Vpu, and Nef counteract several cellular restriction factors to secure efficient replication. Table 1 provides an overview of the best characterized restriction factors.

The mature membrane-enveloped HIV-1 virion is spherical in shape with a diameter of approximately 120 nm. The virion’s lipid bilayer membrane contains, besides several host cell proteins, ~7–35 envelope trimers consisting of gp120-SU and the gp41-TM [23,31,32,33]. Both proteins are encoded in the *env* gene and originate from the Env polyprotein gp160 upon cleavage by the cellular furin-like protease [27]. The membrane envelopes the matrix protein (p17-MA) formed core. The viral capsid is formed by 1000 to 1500 cone-shaped hexameric capsid proteins (p24-CA) [34]. The capsid encapsulates two copies of positive-sense and single-stranded gRNAs stabilized by the nucleocapsid proteins (p7-NC). The mature virion harbors the viral enzymes reverse transcriptase (p66-/p51-RT), protease (p10-PR), integrase (p32-IN), and the accessory protein Vpr that are needed in the maturation process [23,35].

### 2.2. Receptors and Cell Entry

**Figure 2:**
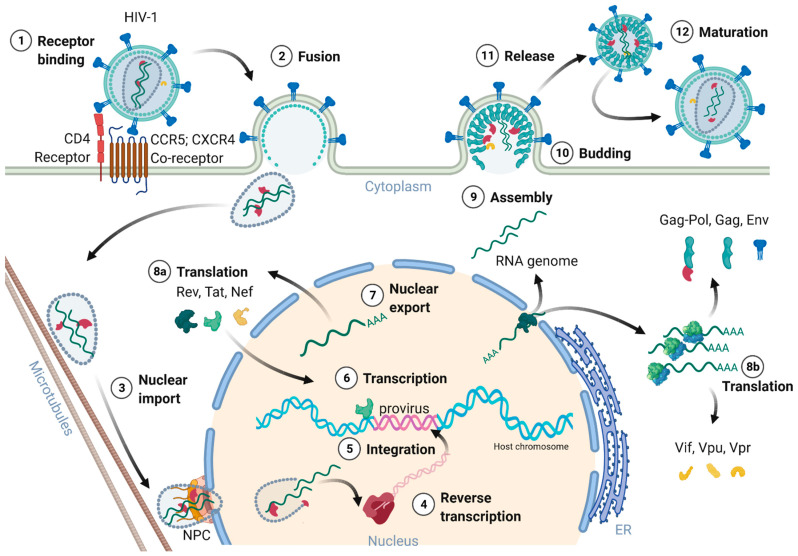
Schematic overview of the HIV-1 replication cycle. (1) The HIV-1 infection begins with the binding of the envelope glycoproteins gp120-SU to the primary CD4 receptor and chemokine co-receptors (CCR5 or CXCR4) on the host cell surface. (2) The virion’s membrane envelope then fuses with the cellular membrane, releasing the viral capsid into the cytoplasm. (3) The capsid travels along the microtubules to the nucleus. The capsid docks to the nuclear pore complex (NPC) and passes through the pore into the nucleus. (4) The capsid partially uncoats during nuclear cell entry and the reverse transcription of the viral gRNA into the provirus is completed inside the nucleus. (5) The integrase together with cellular co-factors promote the integration of the provirus into highly active chromosomal regions of the host genome. (6) Tat activates gene transcription of the provirus. (7) Rev recruits several host proteins to export the intron-containing viral mRNAs. (8a/8b) Viral mRNA translation occurs within the cytoplasm, first Rev, Tat, and Nef are expressed. Signal peptide containing proteins such as Vpu and Env enter the endoplasmic reticulum (ER) for further posttranscriptional modifications. Glycosylated Env passes through the Golgi apparatus and is cleaved by the cellular furin-like proteases into gp120-SU and gp41-TM. (9) Two viral gRNAs, Gag, Pol, Env, and Vpr assemble to nascent HIV-1 particles at the cell membrane. (10) Immature HIV-1 particles bud from the cell membrane. (11) Immature HIV-1 particles are released from the host cell. (12) During maturation, Gag and Pol precursor proteins are cleaved by the viral protease into their subunits MA, CA, and NC as well as the viral enzymes PR, RT, and IN. Upon finalization of the maturation, the newly formed HIV-1 virions are prepared for the next host cell infection, reinitiating a new replication cycle.

Figure 2 provides an overview of the HIV-1 replication cycle. The HIV-1 infection of a host cell is receptor-dependent and begins with the binding of the envelope protein gp120-SU to the primary host cell receptor CD4 and the co-receptors, chemokine receptor type 5 (CCR5), or C-X-C motif chemokine receptor type 4 (CXCR4). The binding induces conformational changes of the envelope protein trimers, which leads to the fusion of the virion with the host cell membrane [36]. In more detail, when Env binds to the co-receptor, the virus exposes the fusion peptide at the N-terminus of gp41-TM, which inserts into the cell membrane. Again, dramatic conformational rearrangements, forming a very stable six-helix bundle, pull both membranes into close proximity, reaching a hemifusion state initiating in a last step the fusion of both membranes [36,37,38]. Although cryo-electron microscopic images of this process exist, many structural aspects of the proteins involved are still not fully understood. Once the fusion pore opens, the virion releases its interior into the cytoplasm of the host cell [39].

### 2.3. Nuclear Entry, Reverse Transcription, and Uncoating

The cone shaped ~60 nm in diameter capsid, consisting of 250 hexamers and 12 pentamers, was believed to partially uncoat or disassemble already within the cytoplasm [20,23,36,40]. However, most recent studies of Zila and colleagues in 2021 provided astonishing insights into the viral capsid and its trafficking along the microtubules of the cell towards the nuclear pore complex (NPC), revealing that the entire capsid enters the nucleus [40]. As the capsid enters the cytoplasm, it travels along the microtubules towards the nucleus aided by dynein and kinesin-1. Next, the capsid docks with its narrow end to the NPC interacting with the NPC-proteins Nup358 and Nup62. Upon nucleoplasm entry, the capsid partially disassembles, releasing the CA interior [40,41]. Dharan and colleagues discovered that the uncoating as well as reverse transcription are completed within the host cell nucleus [41], which was confirmed by two other studies of Burdick and colleagues [42] as well as Müller and co-workers [43] showing that proviral DNA could only be detected inside the nucleus. Therefore, the reverse transcription already starts within the intact capsid and is finalized upon capsid nucleus entry [42,43]. Burdick et al. also discovered that the complete uncoating takes place 1.5 h before provirus integration into the host cell genome and within a range of 1.5 μm proximate to the gene-rich loci in the euchromatin regions.

The reverse transcription of the viral gRNA to proviral dsDNA in infected cells is an important step of the replication cycle. The RNA/DNA-dependent DNA polymerase and RNAse H are part of p66-RT, whereas p51-RT provides conformational stability. The reverse transcription starts with the so-called first strand transfer and the synthesis of the single stranded DNA (ssDNA). The ssDNA is hybridized to the 3′-end of the viral genome and the negative strand DNA synthesis continues. The second strand transfer leads then to the transcription of the positive strand DNA and dsDNA synthesis is finalized [44]. Template switching events and error-prone RT activity contribute to the high genetic variability of HIV [45].

### 2.4. Genome Integration

Retroviruses permanently integrate their reverse transcribed proviruses into the host cell genome, making the virus an everlasting part of the infected host cell. The integrated provirus can remain dormant within the host, and thus escape from the immune system’s detection and response. These properties render HIV to a latent and life-long infection [46].

The proviral integration is mediated by the viral IN in concert with RT [10]. The integrase forms together with the provirus a strong nucleoprotein complex targeting active transcription units for integration into the genome [47]. These units are found in clusters within the less condensed euchromatin characterized by high transcriptional activity. The integration process is divided into two steps. First, the 3′-ends of the provirus is processed and the two terminal nucleotides are removed, exposing a 3′-hydroxyl group and a 5′-overhang. Next, the targeted host DNA is cleaved, and the processed provirus is integrated, ligating the 3′-ends with the 5′-ends of the target DNA [48,49].

### 2.5. Transcription, Splicing, and Protein Expression

After integration of the provirus, it either remains transcriptionally silent and enters latency or initiates the production of new virions. The protein expression of HIV-1 is regulated at the epigenetic, transcriptional, and posttranscriptional level [50,51,52]. Latently infected cells serve as viral reservoirs, resisting eradication during ART and by the immune system due to the absence of target viral protein expression. Latency is induced by infection of resting cells not supporting efficient viral transcription, by inactive proviral integration sites, epigenetic silencing, and by the differentiation of infected effector immune cells to resting memory cells, respectively [52,53]. However, transcription of the provirus and replication can be reactivated.

The HIV-1 provirus utilizes the host transcription machinery. Host transcription factors such as NF-κB, specificity protein 1 (Sp1) and activator protein 1 (AP-1) are known activators of HIV transcription [50,51,54]. General transcription factors, mediator, and RNA polymerase II (RNA Pol II) assemble into the preinitiation complex at the 5′-LTR promoter. The HIV-1 5′-LTR contains three possible transcription start sites (TSS) consisting of three consecutive guanosins (G) at the junction between the R and U3 region. Depending on the TSS used for transcription, the untranslated 5′-region (5′-UTR) of the proviral RNA transcript begins with a single, two, or three G residues [55]. Promoter clearing is mediated by the phosphorylation of the C-terminal domain of RNA pol II mediated by the transcription factor TFIIH [56,57]. A short RNA segment of about 60 nucleotides is transcribed before promoter-proximal pausing occurs. The pausing is triggered by the formation of the TAR RNA stem-loop and the binding of negative transcription elongation factors (N-TEFs) to the preinitiation complex [18,58,59]. The pause is released by Tat binding to TAR, acting as a transcription factor activating positive transcription elongation factor b kinase (P-TEFb) [18,60,61]. In cells, the majority of P-TEFb is part of the 7SK small nuclear ribonucleoprotein (7SK snRNP), in which the catalytic activity of P-TEFb is inhibited by the Hexim-1 protein [62]. McNamara and colleagues suggested a model of Tat-mediated recruitment of the protein phosphatase 1G (PPM1G) to 7SK snRNP to the HIV promoter [61]. PPM1G then dephosphorylates P-TEFb, thus releasing it from the 7Sk snRNP complex. When Tat binds to the released P-TEFb it induces re-phosphorylation. Tat and the activated P-TEFb kinase bind to TAR, bringing the kinase in proximity to the stalled RNA Pol II transcription complex. P-TEFb phosphorylates the C-terminal domain of RNA Pol II and N-TEFs, facilitating the elongation of the viral transcript [18,61,63].

**Figure 3:**
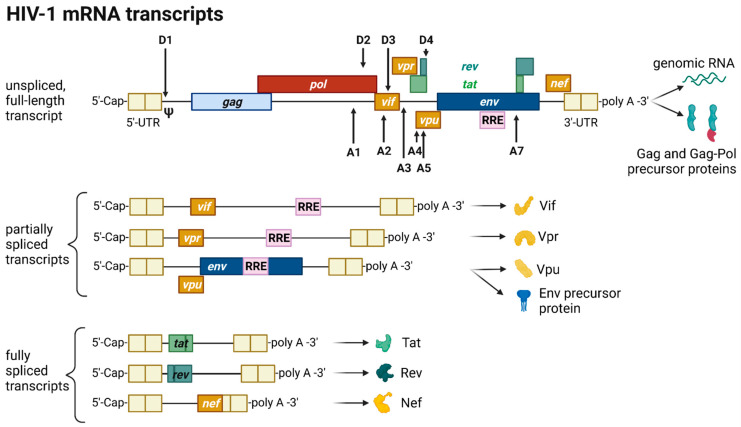
HIV-1 mRNA transcripts and splice sites. HIV-1 transcripts are categorized into three classes: unspliced, full-length genomic gRNA (~9 kb), partially spliced, intron-containing mRNAs (~4 kb) and fully spliced, intronless mRNAs (~2 kb). The class of unspliced mRNAs serves either as gRNA later encapsidated into a virion or as a template for the synthesis of Gag and Gag-Pol precursor proteins. Splicing at splice donor sites (D) to splice acceptor sites (A) generates either partially or fully spliced transcripts depending on the splice sites utilized. All processed HIV mRNAs are spliced at the major splice donor site D1 to a downstream splice acceptor, removing the packaging signal Ψ. In fully spliced mRNAs, the Rev-responsive element (RRE)-containing intron flanked by D4 and A7 is spliced out. The viral proteins Tat, Rev, and Nef are translated from fully spliced mRNAs, whereas Vif, Vpr, Vpu, and the Env precursor protein gp160 are translated from partially spliced transcripts harboring the RRE structure. All transcripts are flanked by untranslated regions (UTR) at the 5′- and 3′-end.

The HIV provirus undergoes three transcription phases [53]: During latency no virions are produced, although stochastic transcriptional bursts at the LTR promoter occur [64]. Upon cell activation, e.g., by immune stimuli, host transcription factors such as NF-κB can reactivate viral transcription and induce the expression of Tat protein, enabling a positive feedback loop. The Tat-mediated transcriptional boost results in the production of full-length gRNA ready to be encapsidated or serving as templates for alternative splicing. The full-length gRNA consists of nine partially overlapping ORFs. Therefore, it is alternatively spliced to generate mRNAs, encoding all viral proteins [14,15,65]. The mRNAs are categorized into three classes: (I) full-length, unspliced ~9 kb gRNA, (II) intron-containing, partially spliced ~4 kb mRNAs, and (III) intronless, fully spliced ~2 kb mRNAs [15,66]. The *gag* and *pol* gene products are translated from the unspliced full-length gRNA, whereas the other viral proteins Nef, Rev, Tat, Env precursor protein, Vpr, Vif, and Vpu are produced from either partially or fully spliced mRNAs. Figure 3 provides an overview on the mRNA classes as well as splice donor and acceptor sites present in the HIV-1 mRNA transcript. All HIV mRNAs that undergo splicing utilize the major splice donor site (D1), which defines the first exon between the 5′-Cap and D1 included in all viral mRNAs [65,66]. The exon defined by D4 and either the splice acceptors A3, A4, or A5 and the final exon between A7 and the poly A tail are additional constitutive exons present in all HIV mRNAs [66]. The full-length gRNA transcript is sequentially spliced, starting at D1 to a downstream splice acceptor site and a prerequisite for further downstream splicing [67]. The packaging signal Ψ is removed, and thus ensures selective full-length gRNAs encapsidation into new virions [68]. Splicing of the viral mRNAs is tightly regulated by the cellular spliceosome. As the splicing of D1 to a downstream splice acceptor is mandatory for all subsequent splice events, suppression of splicing at D1 results in unspliced transcripts [66,67]. Noteworthy, the 5′-UTR of the full-length transcript can adopt different secondary conformations depending on the number of guanosines at the 5′-Cap [69]. RNAs that start with a 1G^Cap^ fold into a structure that masks D1 and favors the formation of RNA dimers, whereas RNAs with 2G^Cap^ or 3G^Cap^ fold differently and expose the D1 site for splicing [55,70]. To generate partially spliced mRNAs, splicing events are regulated by a complex interplay of several splicing regulatory elements that modulate the usage of splice sites [15]. Unspliced and partially spliced mRNAs harbor the intron, spanning from D4 to A7. This is pivotal as this intron contains the RRE indispensable for the Rev-mediated nuclear export of intron-containing mRNAs.

Only intronless mRNAs are exported across the NPC by cellular mRNA export pathways. Consequently, only the fully spliced viral mRNA transcripts are exported to the cytoplasm and translated early in the viral replication cycle, first enabling the expression of Tat, Rev, and Nef proteins. In contrast, incompletely spliced, intron-containing mRNAs are excluded from the nuclear export pathway and degraded [71,72]. Once expressed, Rev is transported into the nucleus, where it accumulates and co-transcriptionally binds RRE present in incompletely spliced viral transcripts mediating nuclear export [71,73]. This way, HIV circumvents the nuclear mRNA degradation of RRE-containing transcripts. Rev recruits the cellular export factor chromosomal maintenance 1 (CRM1), which mediates the RanGTP-dependent export of the Rev:RNA:CRM-1 complex to the cytoplasm [50,71,73].

In summary, viral gene expression is regulated via transcription, splicing patterns, and RNA structures. Early in the viral gene expression only fully processed mRNAs are translated into the accessory protein Nef and the regulatory proteins Tat and Rev. Nef increases viral infectivity by remodeling signal pathways, downregulating the expression of cell surface proteins such as CD4, major histocompatibility complex-I, and activation of viral transcription through NF-κB [74,75]. Tat activates and stimulates transcription of the provirus by interaction with cellular co-factors at the TAR RNA structure. Rev enables the export of RRE-containing incompletely processed RNAs, shifting the viral protein expression to proteins necessary for the production of new virions. The mRNAs encoding the p55-Gag precursor, p160-Gag-Pol precursor, and Vif and Vpr proteins are translated by polysomes in the cytosol [76]. The Gag-Pol precursor proteins are translated from the full-length gRNA by a ribosomal frameshift during translation [77]. The bicistronic *vpu/env* mRNA is translated into Vpu and Env precursor gp160 in the rough endoplasmic reticulum (ER). Inside the ER, the Env precursor gp160 assembles into trimers and travels to the Golgi apparatus, in which gp160 gets glycosylated and cleaved by furin-like proteases into the mature Env glycoprotein complex consisting of the subunits gp120-SU and gp41-TM [78]. Env and Vpu are transported to the plasma membrane via the secretory pathway for incorporation into assembling viral particles [78]. In conclusion, all components needed to initiate virus assembly are now available.

### 2.6. Assembly, Budding, and Virion Maturation

The viral structural Gag precursor protein is sufficient for the formation of new particles. Gag consists of four structural domains separated by protease cleavage sites: the N-terminal MA domain, the CA domain, the NC domain flanked by two spacer peptides (SP1 and SP2), and the C-terminal p6 domain. Each domain performs specific functions during assembly and budding of the viral particle via interactions with viral and cellular proteins and RNAs. The gRNA molecules form a dimer selectively recruited for packaging. Intramolecular and intermolecular interactions of gRNA and Gag polyprotein mediate the selective packaging of the viral genome into assembling particles. The 5′-UTR of the gRNA folds into complex structures consisting of several stem-loops, including the packaging signal Ψ and the dimerization initiation signal (DIS). Recent studies by the Summers group revealed that gRNAs exhibiting a sequestered 1G^Cap^ at the 5′-UTR are preferentially packaged and adopt a dimer competent conformation [55,79,80]. In this conformation, the DIS is exposed and two gRNA molecules dimerize through intermolecular DIS base pairing. The gRNA dimers expose several binding sites located in the DIS and Ψ stem-loops for the interaction with the NC domain of the Gag precursor proteins [81]. Binding of gRNA also promotes the dimerization of Gag by protein-protein interactions [82,83]. The Gag:gRNA complex travels to and is anchored in the plasma membrane through the N-terminal myristoylation signal present in the MA domain. HIV-1 assembles at the cell membrane in specific cholesterol- and phosphatidylinositol-(4,5)-bisphosphate (PI(4,5)P_2_)-rich microdomains called lipid rafts. The targeting of Gag to the membrane is regulated by the electrostatical interaction of the highly basic regions located in the MA domain with PI(4,5)P_2_ and the binding of tRNA^Lys^, which prevents binding of MA to intracellular membranes [84,85,86]. In addition and upon simultaneous binding of PI(4,5)P_2_ and gRNA, Gag folds from a compact to an extended conformation enabling the anchoring of the myristoylation signal to the plasma membrane and initiating the multimerization of Gag proteins [87,88]. Gag and Gag-Pol protein multimerization at the plasma membrane is stabilized by CA-CA and CA-SP1 protein-protein interactions [89]. The assembly of Gag at the plasma membrane also induces the retention of Env trimers at assembly sites mediated by an interaction between the Gag MA domain and the C-tail of the Env protein gp41-TM [78]. In addition to Env, the p6 domain of Gag captures Vpr [90]. The growing Gag multimer bends the membrane and forms a spherical nascent particle still connected to the membrane. However, and for the release of the particle, HIV-1 relies on the cellular endosomal sorting complexes required for transport (ESCRT) machinery [91]. Gag recruits the ESCRT complexes via adaptor proteins, which recognize amongst others the amino acid motifs PTAP and LYPX_(n)_L present in the p6 domain. Tumor susceptibility gene 101 protein (Tsg101) is part of the ESCRT-I complex, binds to the PTAP motif, and forms a supercomplex with ESCRT-II, whereas the adaptor protein apoptosis-linked gene 2-interacting protein X (Alix) recognizes LYPX_(n)_L and interacts with ESCRT-III. The ESCRT-III complex constricts the membrane and catalyzes the release of the immature particle [91].

The viral particle matures and reorganizes its structural proteins, gRNAs, and enzymes, resulting in the formation of an infectious virion. The maturation is initiated by the auto-activation of the PR sequentially cleaving the Gag and Gag-Pol precursor proteins releasing the viral enzymes PR, RT, and IN and the structural proteins p17-MA, p24-CA, and p7-NC [92,93]. The structural changes are mandatory for viral infectivity. The NC protein binds tightly to the gRNA dimer and stabilizes linkage between the two gRNA molecules [81,94]. The CA proteins assemble around the NC:gRNA complex encapsidating the viral genome as well as RT and IN [92]. The processing of Gag into its subunits renders the incorporated Env trimers’ fusogenicity. The HIV-1 virion concludes the productive cell infection and is now armed for a new replication cycle [95].

### 2.7. Cytoxicity of HIV Infection

RNAs are able to cause diseases in many different ways controlling and also disrupting multiple genetic and metabolic pathways in the cell [96]. For example, the transcription of non-coding repeat expansions can lead to toxic RNAs—e.g., the dominantly inherited and multisystemic disease myotonic dystrophy type 1 (DM1), where CTG repeat expansions in the 3′UTR of the DM1 protein kinase (DMPK) gene generate DMPK mRNAs that are trapped in ribonuclear foci, compromising the availability of RNA-binding protein (RBP) levels. RNA foci are believed to sequestrate bound RBPs and result in toxicity [97,98]. Many disease-related genes encode RBPs, where mutated gene products accumulate as aggregates disrupting cellular functions involved in RNA metabolism [99,100]. Mutations in the RBPs, TAR DNA (TARDBP), FUS RNA-binding protein (FUS), Ataxin 2 (ATXN2) as well as EWS RNA-binding proteins (EWSR1) and many more have been shown to greatly influence disease risks, e.g., amyotrophic lateral sclerosis (ALS) and frontotemporal dementia (FDT) [100]. 

**Figure 4:**
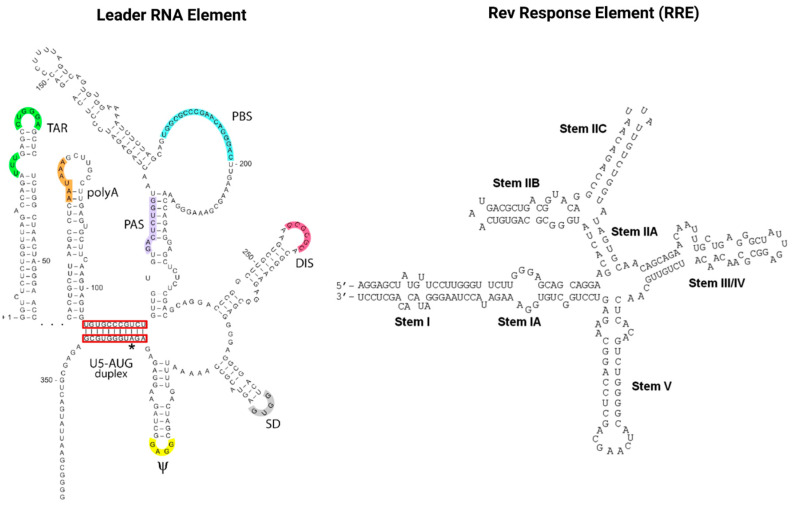
The *cis*-acting RNA regulatory elements of HIV-1. The untranslated highly conserved leader RNA including TAR (left) and the RRE (right). The leader RNA is located in the R and U5 regions of the LTRs of the HIV genome and consists of several regulatory domains: *trans*-activation response element (TAR); polyadenylation hairpin (polyA); primer activation signal (PAS); primer-binding site (PBS); dimerization initiation sequence (DIS); splice donor (SD); RNA packaging signal (Ψ); translation start codon of *gag* (AUG). The highly conserved RRE is located in the *env* gene sequence of the viral genome and contains ~350 nucleotides generating seven stem loops and bulges. Stem IIB and Stem IA are defined as primary and secondary Rev-binding sites (left). Left image adapted from: copyright © 2012, Das et al.; CC BY 2.0 license BioMed Central Ltd. [102]. Right image adapted from: copyright © 2012, Fernandes et al.; CC BY-NC 3.0 license Landes Bioscience [103].

RNAs also play a pivotal role in the HIV infection cycle and pathogenesis. Viral gene expression is regulated *via* transcription splicing patterns and RNA structures interacting with viral and host cell RBPs. Cellular RBPs are strongly recruited away from their cellular functions and cellular cognate target RNAs in response to viral infection, which skews the availability of target RNAs towards HIV transcripts [101]. Maybe most importantly, the two viral regulatory *trans*-acting nuclear RBPs of HIV, Tat and Rev bind *cis*-acting RNA motifs, the TAR and RRE of the newly transcribed HIV genomic RNA, and thus mediate the deregulation of the host cell RNA and protein synthesis machinery to enable efficient virus replication [102,103]. As illustrated in Figure 4, TAR (located in the HIV leader RNA element) and RRE (located in the HIV *env* gene) motifs fold into complex secondary RNA structures folding into highly conserved stem loops and bulges. Rev and RRE are known to assemble to a homo-oligomeric ribonucleoprotein complex needed for the nuclear export of intron containing messenger RNAs from the nucleus into the cytoplasm. RRE as well as TAR are also known as target RNA structures for small molecules intervening the HIV replication cycle. However, until today, little is known about the cytotoxic and disease-causing effects of Rev-RRE in contrast to Tat-TAR [103,104]. 

**Figure 5:**
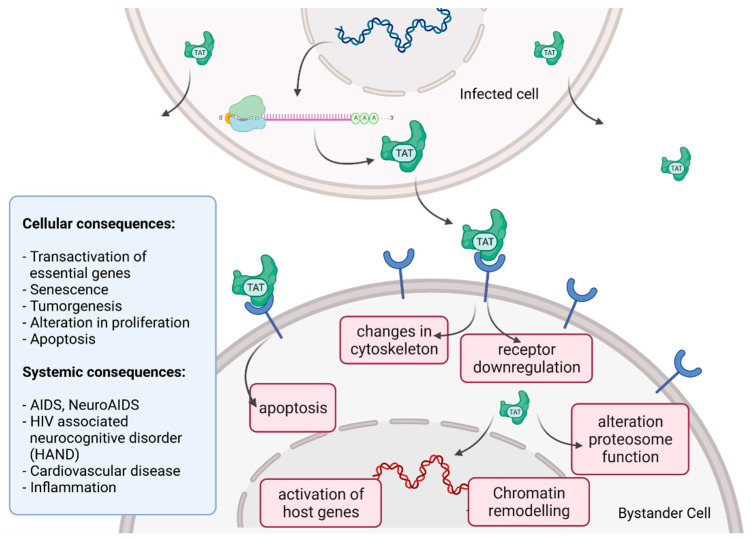
HIV Tat bystander toxicity. Upon infection, Tat accumulates inside the cell but is also released into the extracellular compartment. Tat binds to a range of different surface receptors facilitating the cellular uptake by endocytoses. Chromatin remodeling and transcriptional regulation of gene expression in the nucleus as well as receptor downregulation, changes in the organization of the cytoskeleton and induction of apoptosis can be caused by Tat. Different cellular and systemic alterations are listed (left).

Tat recruits the histone acetyltransferases to the viral promoter to activate the transcription of the viral genome. In addition, the RNA helicase A (RHA) acts as a strong TAR-binding cellular co-factor and enhances HIV-1 LTR-driven gene expression and virus production. The RBP Tat enters the nucleus and binds to the host cell RBP P-TEFb. This complex then interacts with TAR on the RNA enhancing the activity of RNA-Pol II, and thus transcription levels [96,105]. Tat’s role as the *trans*-activator of HIV transcription is fully characterized. Other replication-independent effects mediated by the viral soluble protein Tat cause diseases. Cells constantly release Tat into the extracellular space where it exerts cytotoxicity harming cells in proximity, also known as bystander toxicity, as illustrated in Figure 5 [104]. 

Upon infection, Tat accumulates at the inside of the plasma membrane of infected cells and is released into the extracellular compartment. Tat actively recruits monocytes and macrophages into the areas of infection. By binding to a variety of cell surface receptors, e.g., heparan sulfate proteoglycans (HSPGs), chemokine receptors, integrins and lipoprotein receptor-related protein-1 (LRP-1), Tat is able to penetrate into a range of different cell types, amongst others, monocytes, macrophages, lymphocytes, astrocytes, neurons and cardiomyocytes. Here, Tat induces the release of mainly pro-inflammatory chemokines and cytokines (e.g., CCL2, TNF-α, IL-2, IL-6, IL-8) that activate transmigration and can be toxic to uninfected bystanding cells as cardiomyocytes and the heart. Tat alters the activity of the proteosome complex (e.g., down regulation of cellular proteins and up regulation of viral proteins). As one example, Tat induces the upregulation of Connexin 43 mRNA and proteins in cardiomyocytes and increases lipofuscin levels, a known aging heart biomarker. Tat also leads to the alteration of actin filaments, tight junctions and adhesion molecules, altering the organization of the cytoskeleton. Inside the nucleus Tat recruits RBPs and binds TAR inducing transcriptional regulation of gene expression and chromatin remodeling resulting in many different cellular and systemic alterations [96,104]. In the case of HIV-associated neurocognitive disorder (HAND), Tat can induce neurotoxicity directly as well as indirectly by triggering inflammation through the activation and recruitment of macrophages, microglia and astrocytes into the affected areas of the brain [104]. Latest findings suggest that Tat causes the emergence of neurocognitive and cardiovascular impairments in about 50 to 60% of HIV-infected individuals as a result of Tat’s bystander toxicity [104,106]. 

## 3. Antiretroviral Therapy (ART)

HIV transmission occurs most frequently during sexual contact through exposure to infectious virions penetrating mucosal surfaces [12]. Alternative transmission routes include percutaneous inoculation among drug abusers and intrauterine infection from mother to child during pregnancy. HIV detection is earliest possible approximately 10 days post infection, employing sensitive polymerase chain reaction (PCR) tests [107,108]. The primary infection phase, two to four weeks post infection, can be nearly asymptomatic or is characterized by flu-like symptoms while viral plasma levels typically peak at this phase. In the second phase a decline of plasma viremia results in a chronic establishment of a viral set point, i.e., the individual stable viral load (HIV RNA) of an infected person. The typical CD4^+^ T cell count in a healthy adult amounts to 500 to 1200 cells per μL. During the progression of HIV infection to the occurrence of AIDS, a decline of the CD4^+^ T cell count to <100 cells per µL is observed [107,108]. This progressive loss of CD4^+^ T cells is accompanied by diseases and malignancies in the infected individuals such as opportunistic infections with *Candidia albicans* and *Pneumocystis jirovecii,* resulting in pneumonia or human herpesvirus, causing Kaposi’s sarcoma [109,110]. The majority of untreated infected individuals die after a 10-year latency period.

In 2020, 73% of the 37.6 million HIV-infected individuals had access to antiretroviral therapy (ART) [111]. ART is a combination of three or four antiviral compounds administered in a lifelong treatment regimen [112,113,114]. The therapy does not cure HIV-infected patients but enables the management of HIV infection as a chronic disease. To date, more than 40 antiretroviral drugs categorized in 7 classes are approved by the U.S. Food and Drug Administration (FDA) and recommended for HIV treatment [115]. Table 2 gives an overview of these classes and some exemplary compounds. These antiviral compounds interfere with key steps of the viral replication cycle and comprise the (I) nucleoside reverse transcriptase inhibitors (NRTIs) including the first approved antiretroviral drug zidovudine (Retrovir), (II) non-nucleoside reverse transcriptase inhibitors (NNRTIs), (III) protease inhibitors (PIs), (IV) integrase inhibitors (IIs), (V) (post-)attachment inhibitors (AIs), (VI) CCR5 receptor antagonists, and (VII) fusion inhibitors (FIs). The latter two classes share a similar approach of impeding cell entry. CCR5 antagonists block the cognate co-receptor of CD4^+^ T cells. Blocking of CCR5 consequently prevents the initialization of the gp41-TM-mediated membrane fusion [116]. Fusion inhibitors on the other hand directly block the retroviral entry by gp41-TM fusion peptide binding [117]. The recently approved attachment inhibitors Fostemsavir and the therapeutic antibody Ibalizumab-uiyk prevent retroviral entry by blocking HIV gp120-SU and the CD4 receptor, respectively [118,119]. Drugs from the other four classes (NRTIs, NNRTIs, IIs, and PIs) on the contrary do not target retroviral entry, but inhibit key enzymes within the replication cycle [13,120,121]. NRTIs and NNRTIs both inhibit provirus synthesis by either leading to chain termination during DNA strand elongation or by directly inhibiting reverse transcriptase activity. Integrase inhibitors, on the other hand, prevent the insertion of the synthesized provirus into the host cell genome. Protease inhibitors block the processing of precursor proteins during assembly and maturation of particles to infectious virions [122].

The use of ART clearly improves the prognosis of HIV-infected individuals since the viral load is suppressed to a steadily low level, preventing progressive CD4^+^ T cell decline [123,124]. Moreover, the suppression of the plasma viremia to an almost undetectable level decreases the sexual transmission of HIV sustainably, and thus also facilitates prevention of new infections. The risk to acquire HIV infection within a HIV-discordant relationship is reduced by 96% when ART treatment is initiated immediately or early after HIV diagnosis [125,126]. In addition, a post-exposure prophylaxis (PEP) treatment with antiretrovirals (tenofovir, emtricitabine, and raltegravir) can reduce the transmission risk by 80%. PEP can be initiated shortly after or ideally within the first 72 h after occupational contact (blood or blood-containing fluid) or after non occupational exposure to the virus [127,128]. ART has to be administered in a stringent and lifelong treatment regimen that requires the variation of different drug combinations to avoid the occurrence of drug resistant viruses quickly emerging during monotherapy [123,129,130,131,132]. Since the development and approval of the first antiretroviral drug in 1987, substantial progress in the treatment of HIV infection was achieved [133,134]. The health-related quality of life among HIV-infected individuals has remarkably improved using state-of-the art drugs and advanced dosage schedules [114,123,124,125,126]. For example, the recently approved integrase inhibitor cabotegravir and the NNRTI rilpivirine show extended half-life, and thus can be administered on a monthly basis, hence remarkably improving treatment of people living with HIV [135,136].

To reduce the propensity of re-emerging drug resistant variants, promising targets for compound-mediated therapeutic interventions could include conserved mRNA structures such as hairpins, stem-loops, and bulges present in TAR [137,138,139,140], RRE [62,141,142], and Psi [143,144,145], as these structurers interact specifically with their cognate viral protein counterparts, namely, Tat, Rev, and the p7-NC of the core protein Gag, respectively. However, these novel approaches are still in the pioneering stage.

The use of antiretroviral medicine for pre-exposure prophylaxis (PrEP) became evident as a successful preventive method, despite being associated with high costs and limited access [146]. Positive effects on the reduction of AIDS mortalities resulted from national and global ART campaigns, but in view of slowly decreasing infection numbers and stagnating funding, the ambitious 90-90-90 target is unlikely to be reached [147]. The 90-90-90 target was a strategy based on three pillars, which was announced by the joint United Nations program on HIV/AIDS (UNAIDS) in 2014 claiming that, in 2020 (I) 90% of HIV-infected people will be diagnosed, (II) 90% of those diagnosed will receive ART, and that (III) 90% of those on ART will have a controlled viral load suppression. However, only 5 of more than 40 countries participating in the U.S. President’s Emergency Plan for AIDS Relief Countries (PEPFAR) reached this ambitious goal [148]. The U.S. government orchestrates PEPFAR and thereby supports countries with high HIV prevalence in epidemic control such as Uganda, Rwanda, and South Africa [149]. Reaching the global 90-90-90 target expectation remains difficult to meet. The socioeconomic and geopolitical instable situation, e.g., in the Middle East and North Africa present a constant obstacle and complication for achieving the 90-90-90 target [150]. Much alike and in contrast to Western Europe (84-88-90), a strategy progress monitoring revealed that Eastern Europe (57-45-57) is far away from reaching the target [151].

A quarter of the HIV-infected population worldwide has still no access to ART in 2020, most likely due to infrastructural or financial limitations [111]. Accordingly, HIV treatment in the Western world reveals high lifetime costs of at least USD 326,500 for an individual who acquires HIV at the age of 35 as estimated by a US study from 2015 [152]. The average price of first-line antiretroviral drugs in the US has increased more than 30% since 2012, which is 3.5 times the rate of inflation [153]. Whether the huge financial costs associated with therapy and patient care are manageable in the future appears questionable. A stable health infrastructure is crucial since viral load rapidly rebounds within weeks after ART interruption, supporting the emergence of drug resistant virus variants [154,155,156].

In view of these obstacles, new global initiatives for HIV prevention are required to tackle the challenges and worldwide financial burden of this epidemic [157]. Ideally, a prophylactic HIV vaccine would be available, enabling global vaccination campaigns in the near future.

## 4. Vaccines

### 4.1. Interplay of HIV and Immune Response—Implications for Vaccine Development

A financially sustainable alternative to the current ART is necessary to halt the progression of the HIV epidemic. The development of a vaccine followed by a global vaccination campaign is considered the most effective strategy. However, and over the last decades, the development of a potent vaccine has been unsuccessful [158]. The obstacles for the development of a vaccine are rooted in the unique biology of HIV. The high mutation and recombination rate of the virus generates repeatedly novel immune escape variants [45,46]. In addition, latency facilitates the establishment of viral reservoirs. These two characteristics mainly hamper the design and development of an effective HIV vaccine [159,160,161,162].

Besides dendritic cells and macrophages, CD4^+^ T cells are the main targets of HIV replication. During viremia, infected cells disseminate throughout the body and the viral load increases until hitting a peak after two to four weeks post infection [108]. As part of the cell-mediated immune response, infected CD4^+^ T cells underlie clearance by CD8^+^ cytotoxic T lymphocytes (CTLs), which are subsequently activated upon infection and mostly specific for the Gag proteins of HIV [107,108,163]. The following long-term steady state of low viral load is mainly a result of CTL activity limiting HIV replication [164]. Whereas most HIV-producing cells are eradicated by the immune system in the early phase of infection, small pools of non-activated or naïve infected CD4^+^ T and T memory cells persist, still containing proviruses [53]. This small pool of cells serves as a viral reservoir that remains dormant until provirus expression is initiated upon antigen- or cytokine-mediated activation [53]. In addition, harboring proviruses of CTL escape variants and ensures that these infected cell pools remain unrecognized by the cellular immune system [46,165]. This way, the viral reservoir represents a genetic archive of numerous HIV variants whose vast majority was generated during viremia [166]. Therefore, vaccination must achieve an early and effective CTL activity in order to control and suppress viremia after infection and hence limit the probability of establishing viral reservoirs.

In parallel to the cellular immune response, the humoral immune response is rapidly activated after infection, resulting in the production of HIV-specific antibodies, amongst others, directed against various target epitopes in the Env proteins [167]. However, the vast majority of the Env-binding antibodies target epitopes, not mediating virus neutralization [167,168]. In addition, neutralization-sensitive epitopes are mostly masked by the high density of glycosylation of the Env proteins [169,170]. The resulting glycan shield thus serves as a barrier of virus neutralization by the humoral immune response. Nevertheless, the antibody response still acts as a selection pressure on the virus, leading to the continuous adaptation of Env, and thus the generation of new viral variants evading humoral immune response [168,171]. However, this co-evolution of Env and antibody response also drives the emergence of so-called “broadly neutralizing antibodies” in 20–30% of HIV-1 infected individuals [172]. Broadly neutralizing antibodies (bNAbs) target distinct and highly conserved neutralization-sensitive epitopes on Env trimers [173,174]. BNAbs recognize either proteinaceous epitopes or target glycan structures. These bNAbs also mediate the neutralization of a broad range of HIV variants, whereas most induced neutralizing antibodies are variant- or strain-specific [172,175,176]. Highly potent bNAbs were isolated from HIV-infected individuals [177,178].

Noteworthy, a minority of less than 1% of HIV-infected individuals show low viral loads close to the detection limit of very sensitive PCR-mediated diagnostic assays [179,180]. These low viral loads are correlated with a strong CTL response and a decline of infected CD4^+^ T cells [180]. Individuals exhibiting this trait of spontaneous disease control are summarized under the term “elite controllers” [179,181]. However, the exact mechanism of how elite controllers maintain low viral loads over the years is not yet fully understood despite being of major interest for vaccine design [182]. Some observations point towards an improved Gag-specific T cell response and distinct provirus integration sites [181,183]. This group of HIV-infected individuals therefore represents the closest approximation to how immunity against or control of HIV could be achieved [182,183].

An ideal HIV vaccine would thus likely consist of two components [184,185]. One component should elicit a bNAb response to combat the large Env diversity of globally circulating HIV variants and consequently prevent infection of new host cells. From the viewpoint of vaccine development, the striking variation of Env represents a particular challenge for the design of potent target antigens [186,187,188,189]. Therefore, and to gain a deeper understanding of virus neutralization, the identification and examination of the structure of neutralization-sensitive epitopes became of paramount importance for vaccine development [186,190]. The other component should induce an early and effective T cell response to suppress initial viremia, hence preventing the establishment of viral reservoirs. However, it remains unclear whether a future HIV vaccine will confer sterile immunity or rather facilitate virus replication and viral load suppression, preventing the progression to AIDS and further transmission [191].

### 4.2. HIV Clinical Vaccine Trials

In 1986, Zagury and colleagues initiated the first HIV vaccine clinical phase I trial in the Democratic Republic of Congo [192]. Since then, numerous further efforts were undertaken to develop a potent HIV vaccine. The scientific challenge of developing a prophylactic vaccine has been pursued now for over three decades and is mainly obstructed by the extremely high variability of HIV and constant immune evasion of new virus variants. The lack of ideal animal models allowing for preclinical testing of vaccine candidates and delivering reliable data predictive for the later desired potency in humans further hampers the development process [193,194,195].

Three different aims are usually targeted in HIV vaccine development: (I) elicitation of a potent CTL-mediated immunity, (II) induction of a HIV-specific non-neutralizing antibody response, and (III) generation of bNAbs [185]. Several novel approaches to address these assumed “correlates of protection” were already investigated successfully in non-human primate (NHP) studies but revealing limited efficacies in clinical trials in the past years [196].

In this initial regimen, Zagury and colleagues used a vaccinia vector expressing the unprocessed precursor of the HIV envelope protein (gp160). With this approach, it was aimed to induce neutralizing antibodies directed against Env and a parallel potent CTL response [192,197]. The employed vector-based approach was decisive for subsequent vaccination concepts such as the highly anticipated RV144 trial conducted in Thailand in the millennium. In this trial, participants received an attenuated canarypox vector. The regimen comprised prime injections with the canarypox vector vaccine and two booster injections with a recombinant bivalent gp120-SU subunit vaccine derived from HIV subgroup B/E [198,199]. The resulting immune response involved neutralizing antibodies targeting the V1V2-loop of the gp120-SU and a readily detected CD4^+^ T cell response [200,201,202]. Both were presumably accountable for an observed lower risk of infection [202,203]. The trial demonstrated a 31% efficacy and raised hopes that a prophylactic vaccine could be developed, potentially reaching higher efficacies [198,199]. Due to the moderate success of the RV144 trial in Thailand, the vector and the adjuvanted subunit vaccine components were adapted and applied in different regimens of several follow-up studies such as HVTN 305, HVTN 306, and HVTN 702 [204,205,206].

Initiated in 2012, HVTN 305 utilized a late boost regimen conducted with 162 HIV-negative RV144 vaccinated recipients aiming at the induction of long-lasting antibody responses. Although immune responses were elevated compared to the initial vaccination series, a durable antibody response was not achieved. In addition, the induced antibodies were barely capable of neutralizing sensitive laboratory-adapted tier 1 HIV strains. Tier 2 strains, representing the circulating viruses, were not neutralized at all [204,207]. The second follow-up study, HVTN 306, started a year later and focused on the effect of less frequent booster injections after the initial vaccination series during the RV144 trial. The prolonged intervals between initial vaccination and boosting showed a positive effect on the magnitude and quality of immune responses [205]. A third follow-up study in South Africa (HVTN 702) was launched in 2016 exchanging the gp120-SU antigens derived from clade B/E with the ones of clade C. The vaccine elicited the desired immune response and reached clinical phase III. However, this new vaccine did not prevent HIV infection in the South African participants [206].

The idea of so-called “mosaic vaccines” was developed to combat the genetic diversity of HIV [208]. Mosaic HIV proteins consist of synthetically shuffled epitopes derived from different HIV variants. Fischer and colleagues disclosed the design of such mosaic HIV vaccines in 2007 and since then research teams around Barouch and Santra picked up the idea and tested mosaic vaccines in rhesus macaques [208,209,210,211]. Barouch et al. used a non-replicating adenoviral vector transferring *gag*, *pol,* and *env* mosaic genes. In contrast, Santra et al. administered a DNA vector, containing *gag* and *nef* mosaic genes for priming, followed by booster injections with a recombinant vaccinia virus. Despite the use of different mosaic HIV antigens (Gag, Pol, Env, and Nef) in different regimens and vector systems, both studies revealed a similar positive outcome in rhesus macaques. Compared to natural occurring antigens, the mosaic proteins mediated the enhanced T cell epitope recognition of CD8^+^ and CD4^+^ T cells and the cross-recognition of variants of these epitopes [209,210,211]. Encouraged by these promising results, an adenovirus serotype 26 (Ad26) vectored vaccine Ad26.Mos.HIV (consisting of Ad26.Mos.1.Env, Ad26.Mos1.Gag-Pol, Ad26.Mos2.Gag-Pol), a modified vaccinia Ankara (MVA)-Mosaic vaccine (MVA.Mos.1.Env, MVA.Mos1.Gag-Pol, MVA.Mos2.Gag-Pol) and a subsequent protein boost with adjuvanted clade C gp140 proteins (truncated Env precursors) were tested in a clinical trial (APPROACH) and a rhesus monkey challenge study [212]. The envelope glycoproteins were either applied in a membrane-anchored form displayed on the surfaces of Ad26.Mos.1.Env transduced cells or as soluble gp140 proteins used for boosting. The protein boost used in the APPROACH study was thereby composed of stabilized Env trimers of clade C, so called SOSIP trimers, assumed to be crucial for the elicitation of broadly neutralizing antibodies [213,214]. In summary, the vaccine regimen was highly immunogenic in humans and in primates alike. A 67% protection against infection with a Simian-Human Immunodeficiency Virus (SHIV)-SF162P3) was achieved when rhesus monkeys were subjected to six intrarectal virus challenges, raising hopes for the desired potency in humans [212]. 

In 2017, the vaccine components of the APPROACH study were further used in an efficacy trial in Southern Africa under the study name Imbokodo [212,215]. Imbokodo enrolled 2637 participants in a phase IIb clinical trial. However, the Imbokodo study was recently terminated ahead of schedule due to disappointing efficacy [216]. Yet, there is still hope for the alternative Mosaico trial, started in 2019, which is a related study analyzing the effects of Ad26.Mos.HIV and an adjuvanted clade C gp140 protein vaccination of participants in North America, Latin America, and Europe [217]. Despite advances in the HIV vaccine development, Mosaico and the previously mentioned HVTN 702 were the only two HIV vaccine efficacy trials that enrolled more than 100 participants and reached phase III in the past 10 years. Table 3 gives a detailed overview of these two trials, including the respective vaccine regimen and trial sites.

## 5. Outlook

The extremely high variability of HIV is a challenge for both the further improvement of ART and the development of a prophylactic vaccine. Whereas most current compounds used in ART target viral proteins prone to hyper mutation, mRNA structures such as hairpins and stem-loops can be targeted and due to their conserved structure potentially offer an opportunity to overcome the issue of virus variability. However, ART is cost-intensive, and thus unlikely to be globally applicable and accessible. Therefore, and to fight the global epidemic of HIV, a prophylactic vaccine appears indispensable.

An efficient vaccine against HIV infection facilitating future global vaccination campaigns needs to induce a strong and sustainable cellular and humoral immune response including the elicitation of cross-clade neutralizing bNAbs. The concept of using multiple mosaic antigens appears promising in order to cover the high diversity of globally circulating HIV variants. However, and after three decades of conducting clinical trials, it seems likely that the combination of different vaccine platforms will be required to generate an efficient polyvalent vaccine. This will most likely include novel technologies such as mRNA-, HIV-derived virus-like particle (VLP)-based and viral vectored vaccines using a variety of different donor viruses.

## Figures and Tables

**Table 1:** Selected examples of cellular HIV restriction factors, mechanism of action, and viral counter measures.

Restriction Factor (RF)	RF Mechanism of Action	HIV Counter Reaction	References
APOBEC3G	Encapsidated into virion, induces G-to-A hypermutation during reverse transcription	Vif	[24,25]
IFITMs/IFI16	Excludes viral mRNA from polysome processing, inhibits the protein synthesis	Nef	[26]
SAMHD1	Deoxynucleoside triphosphate triphosphohydrolase 1 activity, prevents reverse transcription	Vpx (only HIV-2)	[25]
SerinC3/5	Incorporated into the virion, inhibits membrane fusion	Nef	[27]
Tetherin/BST-2	Anchors virions on the cell surface of infected cells, inhibits virion release	Vpu	[28,29]
TRIM5α/TRIMCyp/TRIM22	Binds directly to HIV-1 capsids, accelerates uncoating and inhibits reverse transcription	p24-CA variation	[30]

**Table 2:** Overview of FDA-approved antiretroviral medicines including their mechanisms of action, generic names, and approval year amended from HIVinfo.nih.gov, accessed on 21 October 2021 [115].

Antiretroviral Drug Class	Mechanism of Action	Generic Name, Examples	FDA Approval Year
Nucleoside reverse transcriptase inhibitors (NRTIs)	Incorporation of nucleoside or nucleotide analogues by the reverse transcriptase leads to chain-termination of proviral DNA synthesis [13,120,121]	abacir	1998
		emtricitabine	2003
		lamivudine	1995
		tenofovir disoproxil fumarate	2001
		zidovudine	1987
Non-nucleoside reverse transcriptase inhibitors (NNRTIs)	NNRTIs bind the substrate pocket of the reverse transcriptase, hence reducing polymerase activity and impeding proviral DNA synthesis [13,120,121]	doravirine	2018
		efavirenz	1998
		etravirine	2008
		nevirapine	1996, 2001
		rilpivirine	2011
Protease inhibitors (PIs)	Blocking the active site of the viral protease inhibits the processing of the Gag-Pol polyprotein precursor [13,120,121,122]	atazanavir	2003
		darunavir	2006
		fosamprenavir	2003
		ritonavir	1996
		saquinavir	1995
		tipranavir	2005
Integrase inhibitors (IIs)	Blocking the active site of the viral integrase inhibits insertion of the proviral DNA into host cell genome [13,120,121]	cabotegravir	2021
		dolutegravir	2013
		raltegravir	2007, 2017
(Post-)Attachment inhibitors (AIs)	Viral entry is either prevented by binding to gp120-SU (AIs) or by binding the CD4 receptor (post-AIs) [118,119]	fostemsavir	2020
		ibalizumab-uiyk	2018
CCR5 antagonists	Blocking of the co-receptor CCR5 impedes viral entry [116]	maraviroc	2007
Fusion inhibitors (FIs)	Binding to gp41-TM inhibits viral entry [117]	enfuvirtide	2003

**Table 3:** Overview of HIV vaccine phase III clinical trials in the past 10 years with more than 100 participants.

Vaccine Trial	Study ID	Start Year	Target Site	Vaccine Regimen	Outcome
HVTN 702	NCT02968849	2016	South Africa	IM administration of ALVAC-HIV (vCP2438) at months 0 and 1 followed by IM injection of ALVAC-HIV (vCP2438) and bivalent gp120-MF59 adjuvant at a total dose of 200 µg at months 3, 6, and 12	Safe, no serious adverse events were observed, no sufficient protection
Mosaico	NCT03964415	2019	Argentina Brazil Mexico Peru Italy Poland Spain USA	Priming (IM) with Ad26.Mos4.HIV (Ad26.Mos.1.Env, Ad26.Mos.2S.Env, Ad26.Mos1.Gag-Pol, Ad26.Mos2.Gag-Pol) at months 0 and 3 followed by boosting (IM) with Ad26.Mos4.HIV vaccine and adjuvanted bivalent clade C and mosaic gp140 at months 6 and 12	Results not yet available

Abbreviations: intramuscularly (IM), vCP2438 (canarypox vector 2438), adenoviral vector 26 (Ad26).

## Data Availability

Not applicable.
